# Upper limb dysfunction and activities in daily living in idiopathic normal pressure hydrocephalus

**DOI:** 10.1007/s00701-021-04909-w

**Published:** 2021-07-08

**Authors:** Jani Sirkka, Marita Parviainen, Henna-Kaisa Jyrkkänen, Anne M. Koivisto, Laura Säisänen, Tuomas Rauramaa, Ville Leinonen, Nils Danner

**Affiliations:** 1 Department of Neurosurgery, Kuopio University Hospital and Institute of Clinical Medicine – Neurosurgery, University of Eastern Finland, P.O. Box 100, 70029 KYS Kuopio, Finland; 2 Department of Neurology, Neuro Center, Kuopio University Hospital, Kuopio, Finland; 3 Department of Neurology, Institute of Clinical Medicine, School of Medicine, Faculty of Health Sciences, University of Eastern Finland, Kuopio, Finland; 4 Department of Geriatrics/ Internal Medicine and Rehabilitation, Helsinki University Hospital and Department of Neurosciences, Faculty of Medicine, University of Helsinki, Helsinki, Finland; 5 Clinical Neurophysiology, Imaging Center, Kuopio University Hospital, Kuopio, Finland; 6 Department of Applied Physics, University of Eastern Finland, Kuopio, Finland; 7 Department of Pathology, Kuopio University Hospital and Institute of Clinical Medicine – Pathology, University of Eastern Finland, Kuopio, Finland

**Keywords:** Idiopathic normal pressure hydrocephalus, Symptoms, Upper limb motor function, Shunt surgery, Activities of daily living

## Abstract

**Background:**

Idiopathic normal pressure hydrocephalus (iNPH) is a neurodegenerative disease with a characteristic symptom triad of gait disturbance, cognitive decline, and incontinence. Recently, also dysfunctions in upper limbs have been described in iNPH and reported to improve after shunt surgery. We aim to describe the role of upper limb motor function in the clinical assessment of iNPH patients and its influence on activities of daily living (ADL).

**Methods:**

Seventy-five consecutive patients with probable iNPH were studied pre-operatively and at 3 and 12 months after shunt surgery. The pre-operative evaluation included lumbar drainage of cerebrospinal fluid (tap test). Motor functions were assessed in upper and lower limbs with Grooved Pegboard Test (GPT), Box & Block Test (BBT), Total Score of Gait (TSG), and balance test. ADL was assessed with Barthel’s index and cognition in accordance with the Consortium to Establish a Registry for Alzheimer’s Disease (CERAD).

**Results:**

Patients showed improvement in all motor tests and ADL at 3 months after shunt surgery. The improvement remained stable during the 12-month post-operative follow-up. The motor function tests correlated with each other and with ADL.

**Conclusions:**

A 3-month follow-up period after shunt surgery is adequate to show improvement in motor tasks, and a positive outcome will last for at least 12 months. A shunt-responsive dysfunction of upper limb motor performance plays a major role in ADL of iNPH patients. Therefore, we suggest an evaluation of upper limb motor performance to be included in routine evaluation of iNPH patients.

## Introduction

Idiopathic normal pressure hydrocephalus (iNPH) is a neurodegenerative disease with a clinical symptom triad of disturbed gait, declined cognition, and incontinence [1]. The only effective treatment, shunt surgery, is based on diversion of cerebrospinal fluid (CSF) [2, 3].

Disturbed gait is usually the most prominent symptom of iNPH and plays also a major diagnostic role [2, 4]. The role of upper limbs has received considerably less attention despite some early evidence on their involvement in the clinical characteristics of iNPH [5]. In parallel with gait, upper limb motor function has been proven to improve with shunt surgery or after CSF drainage [6–10]. Subsequently, clinical evaluation of iNPH patients has been suggested to be supplemented with testing of upper limbs [11]. However, the effect of upper limb dysfunction on daily living of iNPH patients is unknown and patients may also experience subjective benefits from shunt surgery even if there is no objective improvement on the iNPH grading scale [12].

Therefore, we aim to characterize associations between upper limb motor function, gait, balance, cognition, and activities of daily living (ADL) and to evaluate their responsiveness to shunt surgery.

## Methods

### Study population

**Fig. 1:**
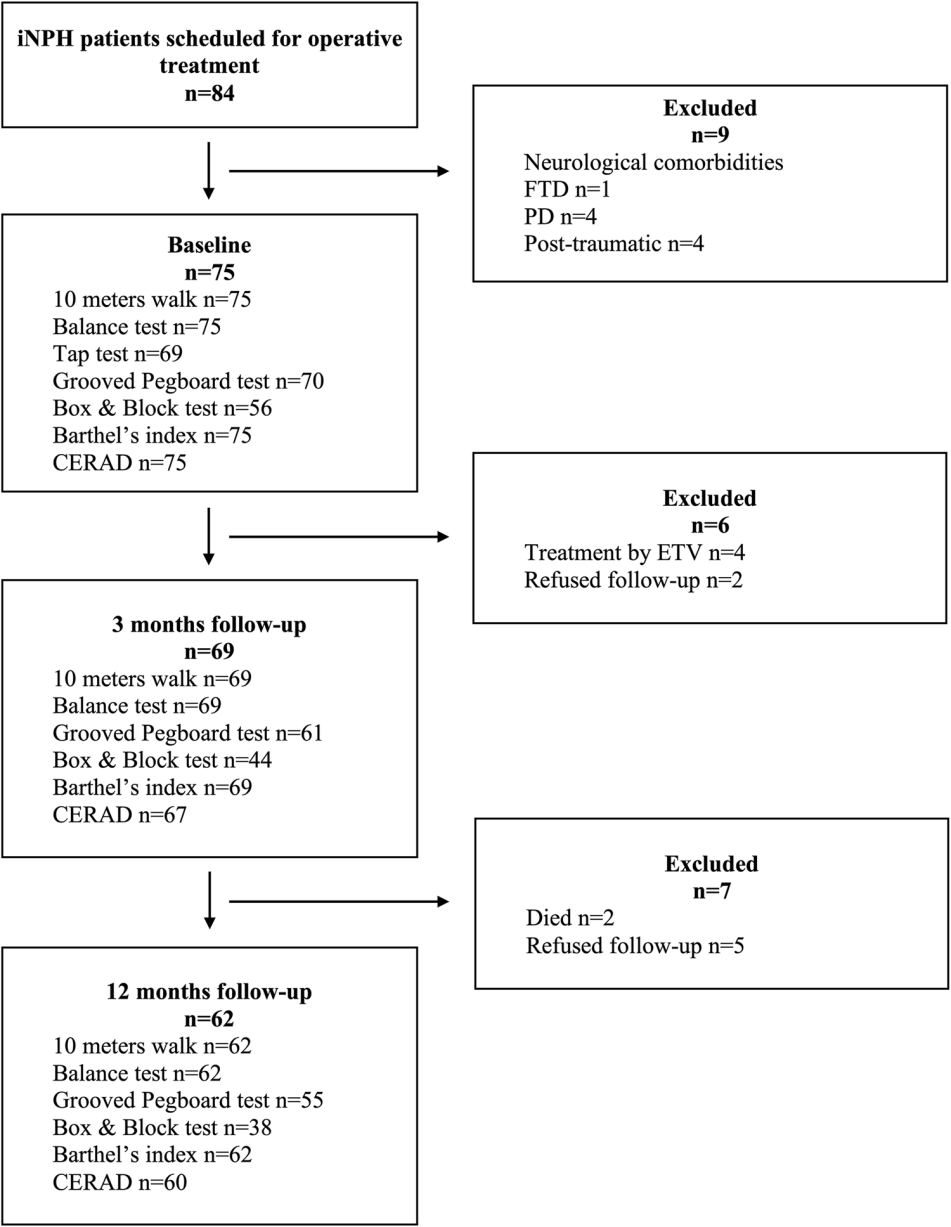
Flow chart of the study ETV Endoscopic third ventriculostomy, FTD frontotemporal dementia, PD Parkinson’s disease, CERAD Consortium to Establish a Registry for Alzheimer’s Disease

The study population was recruited prospectively from the neurosurgical outpatient clinic of Kuopio University Hospital (KUH) from May 2017 to December 2019. All patients had previously undergone a neurological evaluation and were referred to KUH for neurosurgical evaluation due to possible iNPH. Patients had one to three symptoms related to iNPH (impaired gait, declined cognition, or urinary incontinence) together with brain imaging finding of enlarged brain ventricles. During the study period, the total number of patients scheduled for operative treatment was 84 (39 women and 45 men, mean age 75.0 years ± 5.8 years, range 61–86 years). The flow chart of the study is presented in Fig. 1.

### Motor function assessments

Motor function was assessed in accordance with an assessment scale for clinical evaluation of iNPH [13] in three different domains: gait, balance, and upper limb performance. Gait was evaluated by a 10-m walking test, which was repeated three times. The used time, number of steps, and pace were scored from 0 to 100, and then the total score of the gait (TSG) was calculated as an average of these sections. Balance was tested by asking the subject to stand still for 30 s in different positions and scored from 0 to 100. Upper limb fine motor performance was evaluated with the Grooved Pegboard Test (GPT) in which the subject places key shaped pegs in holes with randomly positioned slots. GPT was scored from 0 to 100 depending on the used time. In addition, upper limb gross motor function was tested with the Box & Block Test (BBT). In BBT, the subject moves as many cubes (2.5 × 2.5x2.5 cm) as possible from one box to another within a 60 s time limit [14].

### Activities of daily living and cognition

Activities of daily living (ADL) were assessed with the Barthel’s index (BI) with the following domains (the maximum score of each section is indicated in the brackets): feeding (10), transfers (15), grooming (5), toilet use (10), bathing (5), mobility (15), stair walk (10), dressing (10), bowel control (10), and bladder control (10) giving a maximum score of 100 [15]. An experienced healthcare professional scored the BI by interviewing and observing the ability to function in the above-mentioned sections. The level of cognition was assessed in accordance with the Consortium to Establish a Registry for Alzheimer’s Disease CERAD [16]. The Finnish version of CERAD includes nine subtests: Verbal fluency, 15-Items Boston Naming test, Mini-Mental State Exam (MMSE), Word list learning, Word list recall, Word list recognition, Constructional praxis, Delayed constructional praxis, and Clock drawing [17].

### Tap test

As a part of routine pre-operative evaluation, every patient underwent a tap test in which CSF was drained up to 40 ml by lumbar puncture (LP). The purpose of the tap test was to detect possible immediate improvement in gait which may predict a positive outcome of shunt surgery [18, 19]. The gait test was repeated identically 30–60 min after the LP.

### Surgical treatment

The decision of surgical treatment was made by an experienced neurosurgeon of KUH. The decision was based on the symptom triad, imaging findings and tap test outcome in accordance with the KUH iNPH protocol [12]. In all operated patients, ventriculoperitoneal shunting was performed with an adjustable valve.

### Brain biopsy and immunohistochemistry

During shunt surgery, 1–3 cortical brain biopsies of 2–5 mm in diameter and 3–7 mm in length were obtained using a biopsy needle. The biopsies were taken prior to and using the same route as for placing the ventricular catheter (anterior to the coronal suture and 3 cm from midline). The detailed procedure of the immunohistochemical analysis has been described previously [20]. From all samples, a neuropathologist analyzed the presence of the immunoreactivity for hyperphosphorylated tau (HPτ) and amyloid-beta (Aβ) using light microscopy. Patients were then further categorized into two subgroup by the presence of pathology of the HPτ and/or Aβ and the absence of pathology of the HPτ and Aβ.

### Follow-up

All clinical tests were performed at the outpatient clinic. The baseline tests were performed prior to the TAP test and repeated at 3 and 12 months after shunt surgery (Fig. 1).

### Statistical analysis

Statistical analyses were performed with SPSS (version 24.4; IBM Corporation, Somers, NY). The normality of distribution in each variable was ensured with the Kolmogorov–Smirnov and Shapiro–Wilk tests; CERAD and BBT were normally distributed and other parameters were non-normally distributed. Patients with missing values were excluded from longitudinal and correlation analyses for that parameter (Fig. 1). Baseline and follow-up scores of CERAD and BBT were compared using repeated measures T-test, and parameters with highly skewed distribution (TSG, balance, GPT and BI) were analyzed using Wilcoxon signed-rank test. TSG scores before and after the TAP test were also compared using Wilcoxon signed-rank test. In each test a positive change of one or more points at follow-up was defined as improvement. Linear regression was used to determine differences in shunt surgery outcomes between subgroups. The surgical outcome was defined as a difference between baseline and follow-up scores of each parameter. Age and sex were standardized in linear regression model. Spearman’s test was used for correlation analyses at baseline and follow-ups except for CERAD and BBT for which Pearson’s test was used. *p* values < 0.05 were considered as statistically significant.

## Results

There was significant correlation of upper and lower limb motor function with each other and with cognition (Table 1). At baseline, TSG correlated significantly with GPT and BBT. GPT and BBT exhibited also a significant correlation with each other. BI correlated significantly with all motor function tests and CERAD. CERAD correlated with all motor function tests except balance. At 3-month follow-up after shunt surgery, all parameters correlated with each other. At 12-month follow-up motor function tests except balance and BBT correlated with each other and with CERAD. BI correlated with TSG, balance and CERAD.

**Table 1:** Correlations at baseline, 3-month follow-up, and 12-month follow-up

Baseline					
	**Balance**	**GPT**	**BBT**	**BI**	**CERAD**
**TSG**	0.613**	0.470**	0.435**	0.705**	0.322**
**Balance**		0.522**	0.413*	0.586**	0.217
**GPT**			0.615**	0.482**	0.445**
**BBT**				0.496**	0.306*
**BI**					0.298*
**3-month follow-up**					
	**Balance**	**GPT**	**BBT**	**BI**	**CERAD**
**TSG**	0.657**	0.615**	0.471**	0.566**	0.495**
**Balance**		0.657**	0.500**	0.530**	0.407**
**GPT**			0.721**	0.570**	0.604**
**BBT**				0.670**	0.677**
**BI**					0.503**
**12-month follow-up**					
	**Balance**	**GPT**	**BBT**	**BI**	**CERAD**
**TSG**	0.568**	0.409**	0.482*	0.563**	0.569**
**Balance**		0.404**	0.223	0.334**	0.358**
**GPT**			0.459**	0.197	0.633**
**BBT**				0.240	0.480**
**BI**					0.332**

^*^p < 0.05 and **p < 0.01

BBT = Box & Block test, GPT = Grooved Pegboard test, TSG = Total score of gait, BI = Barthel’s index

**Fig. 2:**
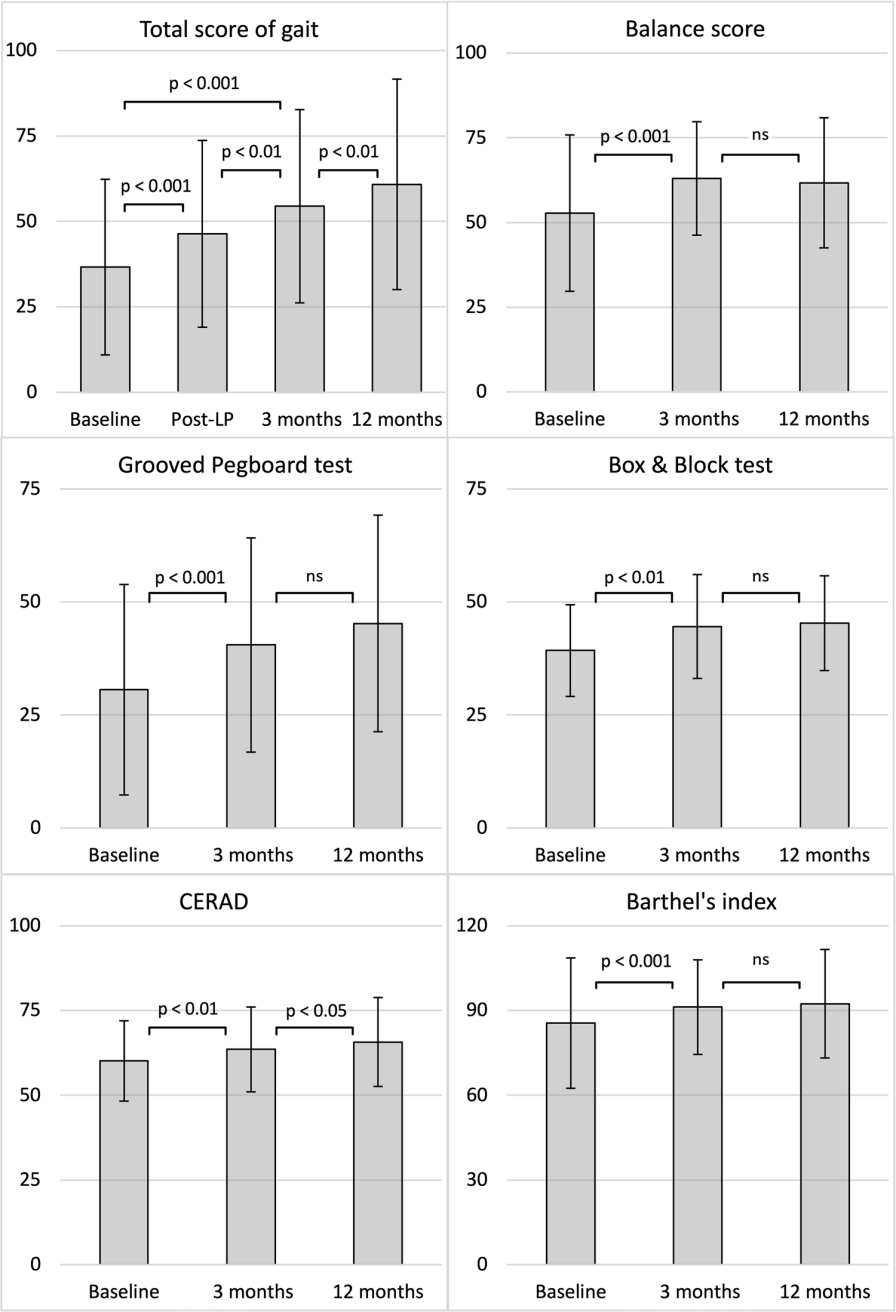
Scores of individual tests at baseline and at follow-ups means and standard deviations of test scores at baseline and at 3- and 12- month follow-ups after shunt surgery

**Fig. 3:**
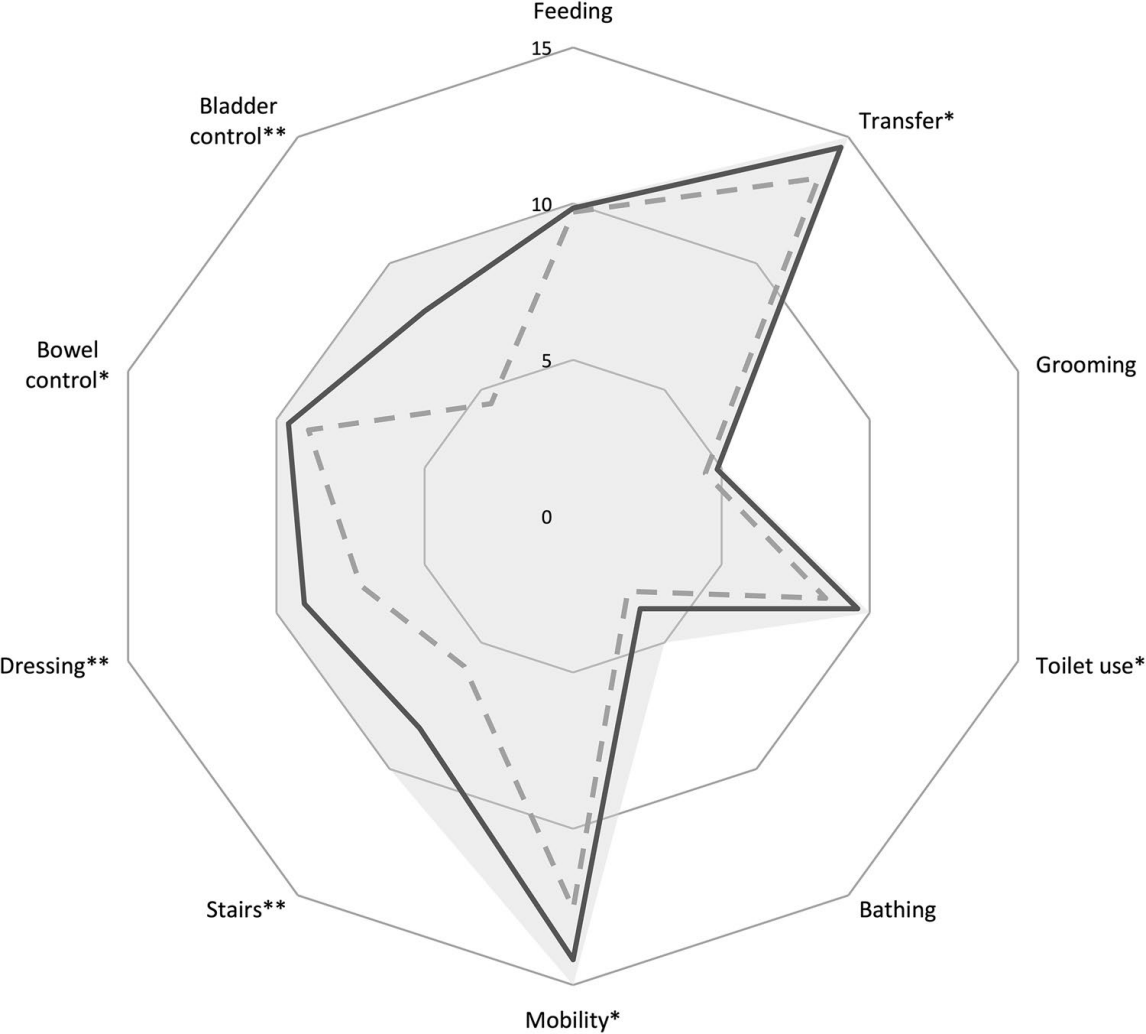
Domains of Barthel’s index in patients with improved total score the grey dotted line represents the mean of baseline values. The black solid line represents the mean of three-month follow-up values. The grey area represents the scale of Barthel’s index, *p < 0.05 and **p < 0.01 (difference between baseline and three-month follow-up)

In the tap test TSG improved in 52/65 (80.0%) patients (*p* < 0.001) (Fig. 1). The improvement correlated with the final TSG at follow-up (r = 0.367, *p* = 0.003) but not with changes in GPT or in BBT. Three months after shunt surgery, the TSG score was higher than after the tap test (*p* = 0.003), and at 12-month follow-up it increased even further (*p* = 0.005). In addition, balance score (*p* < 0.001), GPT (*p* < 0.001), BBT (*p* = 0.002), BI (*p* < 0.001), and CERAD (*p* = 0.002) were significantly higher at 3-month follow-up after shunt surgery than at baseline (Fig. 2 and Table 2). Improvement rates of test scores at 3-month follow up are shown in Table 2. Changes in each BI domain are illustrated in Fig. 3. Cortical brain biopsy was obtained from 63 patients. Thirty-three patients exhibited normal findings in the immunohistochemical analysis, whereas 30 patients had Aβ and 7 patients had HPτ. There were no significant differences in outcomes of shunt surgery between patients who had cortical brain pathology (Aβ and/or HPτ) and those who had not (Table 2).

**Table 2:** Summary of shunt surgery outcomes

	**Baseline**	**3 months **	**12 months **	**Improvement rate**^**a**^
**TSG**	36.7 ± 25.7	54.5 ± 28.3	60.9 ± 30.8	85.6% (59/69)
Biopsy+	32.2 ± 24.1	46.9 ± 23.7	53.9 ± 26.8	82.8% (24/29)
Biopsy-	41.7 ± 28.4	60.3 ± 30.7	67.4 ± 32.3	84.4% (27/32)
**Balance**	52.8 ± 23.1	63.0 ± 16.8	61.7 ± 19.2	39.1% (27/69)
Biopsy+	46.8 ± 26.1	60.6 ± 22.0	57.6 ± 22.8	48.3% (14/29)
Biopsy-	54.8 ± 21.4	64.8 ± 12.8	63.3 ± 16.3	40.6% (13/32)
**GPT**	30.6 ± 23.3	40.5 ± 23.7	45.3 ± 23.9	57.4% (35/61)
Biopsy+	30.7 ± 22.7	38.9 ± 21.9	42.0 ± 21.6	48.1% (13/27)
Biopsy-	29.7 ± 23.7	42.2 ± 24.4	47.9 ± 24.5	66.7% (18/27)
**BBT**	39.3 ± 10.1	44.6 ± 11.5	45.3 ± 10.5	72.7% (32/44)
Biopsy+	40.2 ± 9.7	45.0 ± 10.1	45.7 ± 10.2	70.8% (17/24)
Biopsy-	37.2 ± 11.3	44.4 ± 14.1	45.2 ± 12.0	81.3% (13/16)
**BI**	85.6 ± 17.3	91.2 ± 13.4	92.4 ± 11.2	49.3% (34/69)
Biopsy+	80.3 ± 22.3	90.2 ± 14.5	91.7 ± 8.7	55.2% (16/29)
Biopsy-	88.8 ± 11.9	91.6 ± 14.2	93.3 ± 13.2	40.6% (13/32)
**CERAD**	60.1 ± 11.9	63.5 ± 12.5	65.7 ± 13.1	64.2% (43/67)
Biopsy+	58.5 ± 11.5	62.1 ± 11.4	63.0 ± 13.4	64.3% (18/28)
Biopsy-	61.6 ± 13.1	64.4 ± 14.2	68.6 ± 13.1	59.4% (19/31)

^a^Improvement of one or more points in the test score at 3-month follow-up

Biopsy+ = subgroup with hyperphosphorylated tau and/or amyloid-beta (Aβ) in cortical brain biopsy

Biopsy- = subgroup without hyperphosphorylated tau or amyloid-beta (Aβ) in cortical brain biopsy

BBT = Box & Block test, GPT = Grooved Pegboard test, TSG = Total score of gait, BI = Barthel’s index

Means and standard deviations of test scores at baseline and at 3- and 12- month follow-ups after shunt surgery

At baseline, three patients were not able to complete the walking test and two patients were not able to perform GPT. However, even at baseline upper limb function could be evaluated in all patients using BBT. After shunt surgery, all but one patient managed to complete the walking test, and all patients were able to perform GPT.

## Discussion

This prospective cohort study aimed to extensively characterize the motor performance of iNPH patients and the response to shunt surgery. We found that the motor impairment in iNPH is not limited to the classically reported gait problems but extends to comprehensive motor impairment also in the upper limbs. In tandem with gait, the impairment in upper limb motor function seems to be reversible in nature and to respond well to shunt surgery. Furthermore, the motor performance of iNPH patients seems to be closely related to ADL functions.

### Follow-up time

Current iNPH guidelines recommend assessing short-term outcomes of shunt surgery for up to 12 months [21, 22]. In our study population, an improvement in all the applied motor function tests was observed at the first follow-up 3 months after shunt surgery. Of all studied parameters, only CERAD and TSG showed slight further improvement between the 3- and 12-month follow-ups. This indicates that a 3-month follow-up is sufficient to show the potential of improvement after shunt surgery. Most importantly, no worsening of the motor symptoms or cognitive decline was observed during 1-year follow-up.

### Brain pathology

Interestingly, the presence of HPτ and/or Aβ in cortical brain biopsy had no impact on the surgical outcome with regard to motor function, cognition, or ADL. Alzheimer disease-related pathology in brain biopsy (HPτ and Aβ) seems to be frequent in iNPH and should not been used to exclude patients from shunt surgery [23]. This group of patients should be followed up in clinical studies in order to determine, whether the prognosis differs from classical iNPH.

### Upper limb testing

In previous studies, an improvement of upper limb motor function in GPT has been reported at 3 months after shunt surgery and found to remain stable during a 12-month follow-up [11, 24]. Our current results support these findings. However, we found a significant improvement in BBT, a novel test in iNPH, in an even higher proportion of patients than in the previously reported GPT. BBT is used to assess simple gross-motor manual dexterity, and compared with GPT, it does not require as complex and accurate psychomotor and visual performance [25]. Therefore, it may be a more suitable test for patients with severe symptoms. The present study population included three patients with extremely severe motor symptoms who could not walk at all and two patients who could not perform GPT at baseline. Nevertheless, every patient was able to complete the BBT. In addition, unlike in BBT, there is a floor effect in the 10-m walking tests and GPT, commonly used in iNPH, which complicates their usage in extremely poor performing subjects [7].

### Preoperative evaluation

In the preoperative assessment, up to 80% of patients exhibited a positive tap test result (improvement) in TSG, and the improvement rate was even higher at 3 months after shunt surgery. Thus, the tap test result in TSG seems to be highly indicative of the improvement in TSG after shunt surgery. Some previous studies suggest that CSF drainage via LP might also improve upper limb motor function [6, 7]. The present results show a significant improvement in upper limb motor function after shunt surgery. However, the improvement of TSG in the tap test is not associated with improvement in GPT or in BBT after shunt surgery. This indicates that there could be improvement in upper limb motor function even if gait does not improve in the tap test. On the other hand, it has been shown that manual dexterity might not improve acutely after LP [26, 27]. Therefore, the predictive role of upper limb motor function tests as a part of the preoperative TAP test is controversial and not generally used in clinical practice [28, 29]. The underlying dysfunction and the effect of CSF drainage may differ between upper and lower limbs as suggested in our recent study, in which an initial change in corticospinal excitability after decreased intrathecal pressure was associated with improved walking speed but not with upper limb function [30].

### Activities of daily living

In the present study, ADL functions were good in most patients at baseline but still showed improvement at the 3-month follow-up after shunt surgery, and the good outcome lasted for at least 12 months. Expectedly, improvement in the BI was mainly observed in domains related to gait, such as mobility or stair walking and domains of continence. Interestingly, dressing seemed to be among the domains with the highest improvement, which is likely to reflect the motor function of the upper limbs. The BI was also highly associated with all motor function tests both at baseline and at follow-up, which emphasizes the role of motor symptoms in the ADL functions of iNPH patients. The improvement in ADL with shunt surgery is an invaluable result, since patients with iNPH have been reported to function worst as compared with other types of adult hydrocephalus [31]. Even a small improvement of ADL functions might improve the quality of life in patients with iNPH [32], which has been reported even in the absence of objective benefit of shunt surgery in other clinical tests [33]. One previous study has also demonstrated that the improvement in ADL functions could last up to 5 years in iNPH patients with a favorable clinical or subjective outcome of surgical treatment [34]. Hence, improving and maintaining ADL functions with proper treatment is meaningful for patients, their relatives, and even from a socioeconomic point-of-view [35].

## Conclusions

This is the first study to evaluate upper limb motor function with BBT and to characterize the relationship between various motor parameters and ADL functions. A positive shunt response was observed in gait, balance, upper limb motor functions, ADL and cognition. ADL functions correlated strongly with both upper and lower limb motor function. Patients with comorbid non-iNPH-related walking difficulties may especially benefit from using upper limb function in the preoperative evaluation for shunt surgery. It remains to be shown, whether a sub-population of iNPH patients might benefit from shunt surgery in terms of upper limb motor performance even if the preoperative workup does not imply a positive effect on gait. We suggest that upper limb motor function testing should be included in routine pre-operative evaluation and post-operative follow-up of iNPH patients.

### Limitations and generalizability

Despite the study setting of a prospective cohort with very limited exclusion criteria, the patients were able to perform surprisingly well in ADL based on the BI scores. Thus, the results cannot be unquestionably generalized to severely affected individuals. Of the applied parameters only TSG and GPT have been validated as a part of the clinical assessment scale of iNPH [13]. Upper limb motor function was assessed at baseline and at postoperative follow-ups but the role of BBT and GPT as part of preoperative TAP test remains to be elucidated. Due to the clinical setting of the study, all tests could not be carried out in all patients at every time point. The follow-up period of the current study was limited to 1 year. Future studies are warranted to show the longevity of the shunt response in upper limb motor performance.

## Abbreviations

iNPH: Idiopathic normal pressure hydrocephalus
CSF: Cerebrospinal fluid
ADL: Activities of daily living
ETV: Endoscopic third ventriculostomy
FTD: Frontotemporal dementia
PD: Parkinson’s disease
KUH: Kuopio University Hospital
TSG: Total score of the gait
GPT: Grooved Pegboard test
BBT: Box & Block Test
BI: Barthel’s index
LP: Lumbar puncture
HPτ: Hyperphosphorylated tau
Aβ: Amyloid-beta
ns: Non-significant difference
